# Comparison between Arch Zones in Modified Frozen Elephant Trunk Procedure for Complex Thoracic Aortic Diseases

**DOI:** 10.21470/1678-9741-2019-0398

**Published:** 2020

**Authors:** Mustafa Akbulut, Adnan Ak, Ozgur Arslan, Arzu Antal Dönmez, Serpil Taş, Davut Cekmecelioglu, Mesut Sismanoglu, Mehmet Altug Tuncer

**Affiliations:** 1 Department of Cardiovascular Surgery, Kosuyolu Kartal Training and Research Hospital, Istanbul, Turkey.

**Keywords:** Hospital Mortality, Blood Vessel Prosthesis Implantation, Survival Rate, Follow-Up Studies, Aneurysm, Dissecting, Aortic Diseases, Spinal Cord Ischemia, Aorta

## Abstract

**Introduction:**

The aim of this study is to compare postoperative outcomes and follow-up of two different modifications facilitating surgical technique of frozen elephant trunk (FET) procedure for complex thoracic aortic diseases - zone 0 (fixation with total arch debranching) and zone 3 (fixation with islet-shape arch repair).

**Methods:**

From May 2012 to December 2018, data were collected from 139 patients who had been treated with FET procedure for complex thoracic aortic diseases. According to Ishimaru arch map, patients with proximal anastomotic site of hybrid graft at zone 0 and zone 3 were grouped as Group A (n=58, 41.7%) and Group B (n=81, 58.3%), respectively. Mean age of study population was 54.7±11.4 years, and 111 patients were male (79.9%).

**Results:**

In-hospital mortality was observed in 20 (14.4%) patients (n=12, acute type A aortic dissection, and n=4, previous aortic dissection surgery). There was no significant difference between both groups in terms of in-hospital mortality. Four patients from Group A and three patients from Group B had permanent neurological deficit (*P*=0.32). Three patients from both groups had transient spinal cord ischemia (*P*=0.334). Although mean total perfusion time was longer in Group A, duration of visceral ischemia, when compared with Group B, was shorter (*P*<0.001). Five-year survival rate was 82.8% in Group A and 81.5% in Group B (*P*=0.876).

**Conclusion:**

FET procedure is a feasible repair technique in the treatment of complex aortic diseases, providing satisfactory early results. Because of its advantageous aspects, zone 0 fixation with debranching is the preferred technique in our clinic.

**Table:** 

Abbreviations, acronyms & symbols				
ANOVA	= Analysis of variance		CTBAD	= Chronic type B aortic dissection
ASCP	= Antegrade selective cerebral perfusion		EF	= Ejection fraction
ATAAD	= Acute type A aortic dissection		FET	= Frozen elephant trunk
ATBAD	= Acute type B aortic dissection		ICU	= Intensive care unit
AVR	= Aortic valve replacement		LSA	= Left subclavian artery
CABG	= Coronary artery bypass grafting		MR	= Mitral reconstruction
CAD	= Coronary artery diseases		MVR	= Mitral valve replacement
COPD	= Chronic obstructive pulmonary diseases		SCI	= Spinal cord ischemia
CPB	= Cardiopulmonary bypass		SD	= Standard deviation
CT-A	= Computerized tomography angiography		TAA	= Thoracic aortic aneurysms
CTAAD	= Chronic type A aortic dissection		TEVAR	= Thoracic endovascular repair

## INTRODUCTION

The evolution of the treatment of complex aortic diseases began after the ‘elephant trunk procedure’, described by Brost, and hybrid repair methods with lower mortality rates replaced conventional surgery^[1]^. Afterwards, Kato et al.^[2]^ took the first steps of one-stage treatment by implantation of the stent graft through anterograde route and this method had been evolved into the ‘frozen elephant trunk’ (FET) procedure after various modifications in time^[3-6]^. According to the aortic arch mapping system described by Ishimaru and Mitchell, direction of FET modifications was drawn^[7]^. In the classical application of FET procedure, surgeons from Western countries generally seem to prefer total arch replacement in an islet-shape, while Asian colleagues usually prefer supra-aortic de-branching. Initially, the surgeons used to anastomose the proximal portion of the stent graft to the distal of the left subclavian artery at zone 3, in order to ensure longer segment stabilization of descending aorta^[8]^. As with the hybrid arch repairs, surgeons have tried variations of the zone and aortic arch repair to provide technical convenience and increase the benefit to the patients^[9-11]^. In our study, we compared the postoperative outcomes and follow-up of the complex thoracic aortic diseases treated with FET procedure, using either fixation zone 0 with total arch de-branching, or fixation zone 3 with islet-shape arch repair.

## METHODS

### Patients’ Profile

From May 2012 to December 2018, the data of 139 patients who had been treated with FET procedure due to complex thoracic aortic diseases were collected. According to Ishimaru arch map, the patients with proximal anastomotic site of the hybrid graft at zone 0 and zone 3 were grouped as Group A and Group B, respectively. All the patients with any pathological involvement, such as dissection, aneurysm, or occlusion at their supra-aortic branches, were strictly included in Group A. The remaining patients received one of the two surgical modifications, depending on the surgeon’s experience and perioperative preference upon observing the aortic intimal quality. The Research Ethics Committee of our institute approved this retrospective study (No. 2017.6/8-53). The mean age of the study population was 54.7±11.4 years, and 111 patients were male (79.9%). Data were collected for patients’ demographics, indications for intervention, risk factors, procedures, and outcomes. The follow-up protocol included postoperative computerized tomography angiography (CT-A) before discharge, a clinical examination, and a CT-A three months postoperatively and annually thereafter. Emergency operation was performed in 64 patients, who applied with acute type A aortic dissection (n=61), acute complicated type B aortic dissection (n=10), and ruptured thoracic aortic aneurysm (n=1). The preoperative clinical details and patient characteristics are given in Table 1. Twenty-seven patients (19.4%) had a history of previous cardiac operation and 21 of them were for acute type A aortic dissection. There were more patients with previous cardiac surgery in Group A and more patients with positive family history in Group B. There were no differences in the remaining preoperative characteristics between the groups.

**Table 1:** Patients’ characteristics.

	Overall, n (%)	Group A, n (%)	Group B, n (%)	*P*-value
Age, years (mean±SD)	54.7±11.4	54.3±9.35	55.1±12.70	0.68
Male	111 (79.9)	46 (79.3)	65 (80.2)	0.89
Emergency < 24h	72 (51.8)	29 (50)	43 (53.1)	0.72
CAD	25 (18)	13 (22.4)	12 (14.8)	0.25
EF < %50	15 (10.8)	6 (10.3)	9 (11.1)	0.88
Valve disease				
Aortic	18 (12.9)	8 (13.8)	10 (12.3)	0.8
Mitral	7 (5)	2 (3.4)	5 (6.2)	0.69
Previous cardiac surgery	27 (19.4)	17 (29.3)	10 (12.3)	0.012*
Hypertension	124 (89.2)	51 (87.9)	73 (90.1)	0.68
COPD	24 (17.3)	8 (13.8)	16 (19.8)	0.35
Diabetes mellitus	13 (9.4)	5 (8.6)	8 (9.9)	0.8
Creatinine > 2 mg/dl	12 (8.6)	2 (3.4)	10 (12.3)	0.07
History of stroke	5 (3.6)	2 (3.4)	3 (3.7)	1
Marfan disease	6 (4.3)	3 (5.2)	3 (3.7)	0,69
Family history	10 (7.2)	1 (1.7)	9 (11.1)	0.04*
Complex thoracic aortic diseases				
ATAAD	61 (43.9)	28 (48.3)	33 (40.7)	0.377
CTAAD	26 (18.7)	17 (29.3)	9 (11.1)	0.07
ATBAD	10 (7.2)	1 (1.7)	9 (11.1)	0.045*
CTBAD	19 (13.7)	3 (5.2)	16 (19.8)	0.022*
TAA	23 (16.5)	9 (15.5)	14 (17.3)	0.782

* Statistically significant

ATAAD=acute type A aortic dissection; ATBAD=acute type B aortic dissection; CAD=coronary artery diseases; COPD=chronic obstructive pulmonary diseases; CTAAD=chronic type A aortic dissection; CTBAD=chronic type B aortic dissection; EF=ejection fraction; SD=standard deviation; TAA=thoracic aortic aneurysms

### Definitions

The patients who underwent surgery within the first 14 days from the beginning of back pain and other symptoms of dissection were considered as acute dissection. Early outcomes were accepted as complications in 30-day and in-hospital mortalities. Thoracoabdominal CT-A was utilized in diagnosis and follow-up of aortic pathologies. Spinal cord ischemia (SCI) was defined as new-onset transient or permanent paraparesis or paraplegia after surgery. Renal failure was defined as the need for hemodialysis, and respiratory failure was defined as the need for re-intubation or tracheostomy postoperatively.

### Surgical Techniques

In our study, the E-vita Open Plus (JOTEC^®^ GmbH, Germany) prostheses were used in all cases. As previously described^[12]^, central venous catheter, arterial monitorization at left arm, and near-infrared spectroscopy (or NIRS) were used routinely in all patients. Aortic arch repair was performed at moderate hypothermia using bilateral selective antegrade cerebral perfusion (flow rate = 10-15 mL/kg/min). Median sternotomy was performed in all patients. While right subclavian artery was used for direct arterial cannulation, venous drainage was provided through right atrium. A venting cannula was placed through upper-right pulmonary vein. Myocardial protection was ensured with blood cardioplegia. Proximal aorta repair was done during cooling phase. After aortic clamp was removed, selective antegrade cerebral perfusion was initiated. E-vita Open Plus prosthesis was introduced and released in an antegrade fashion into the true lumen of the descending aorta over the wire with the guidance of transesophageal echocardiography and it was fixed to the aorta with single U-sutures at the selected zone. Stent graft size was determined by measuring the actual lumen diameter of aorta in preoperatively performed CT-A imaging. Two different anastomotic techniques were used when aortic arch replacement was performed:

1. If the proximal suture line of the FET graft was at zone 0, all branches were separated and ligated at ostial level. Perioperatively prepared handmade four-branched graft was anastomosed to the proximal end of the FET graft and then body warming was initiated by placing an additional arterial line to one of the branches. ‘Total arch de-branching’ was performed by anastomosing separate grafts with six or seven individual pledgeted 4/0 prolene sutures (Figure 1). The arterial cannula in the right subclavian artery was withdrawn after all anastomoses were completed.
  **Fig. 1:**
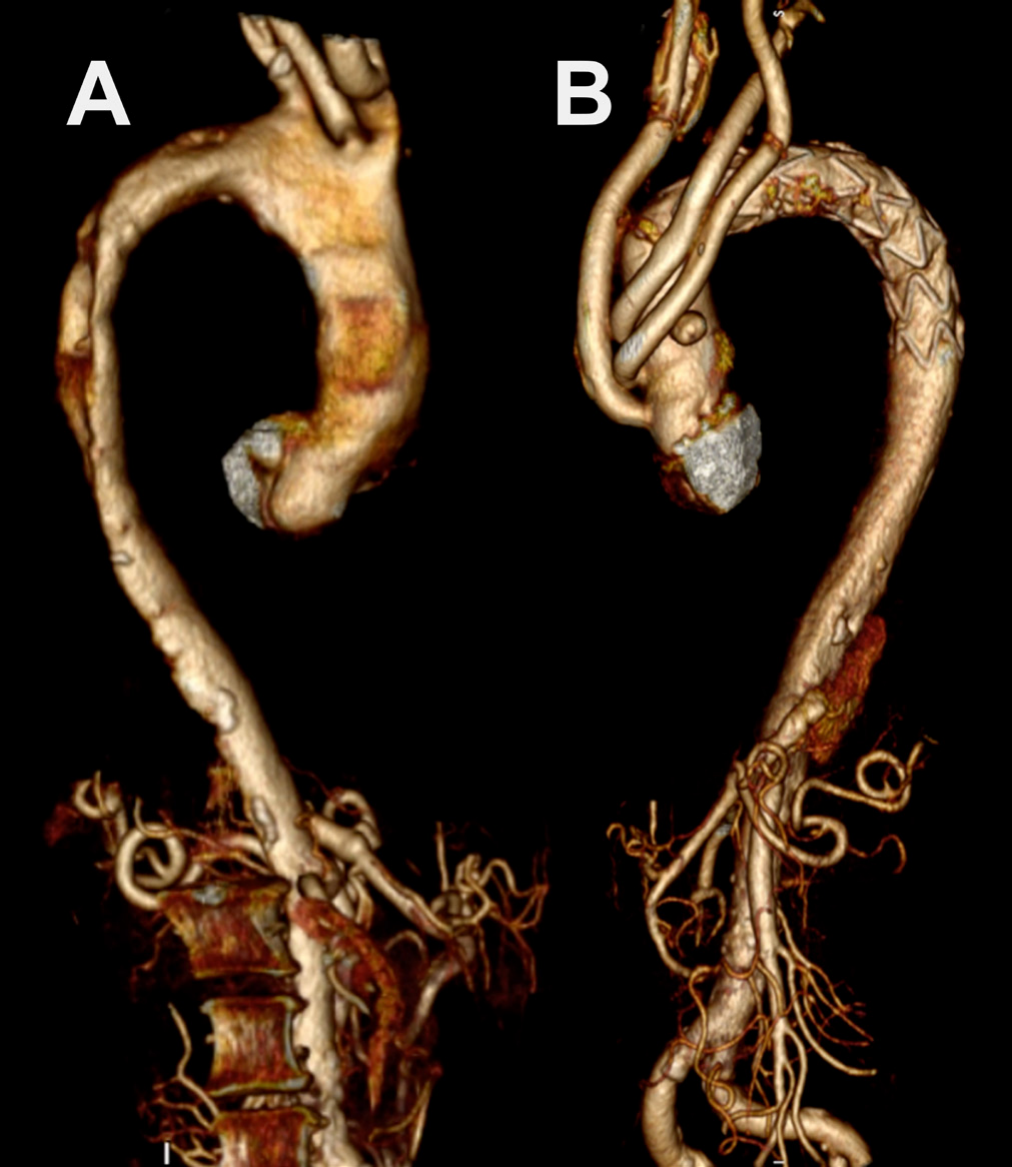
Preoperative (A) and postoperative (B) computerized tomography angiography images of frozen elephant trunk modification; zone 0, fixation with total arch de-branching.
2. In patients with proximal anastomosis of FET grafts performed at zone 3, we used the ‘light arch replacement’ technique that was previously described by Gorlitzer et al.^[13]^. Instead of isolating the supra-aortic branches and extracting them in the form of islets and sewing them *en bloc*, this technique creates a rim without breaking the connection of the aortic arch with the descending aorta and provides an anastomosis similar to hemiarch replacement with a Dacron graft using a running mattress suture (Figure 2).
  **Fig. 2:**
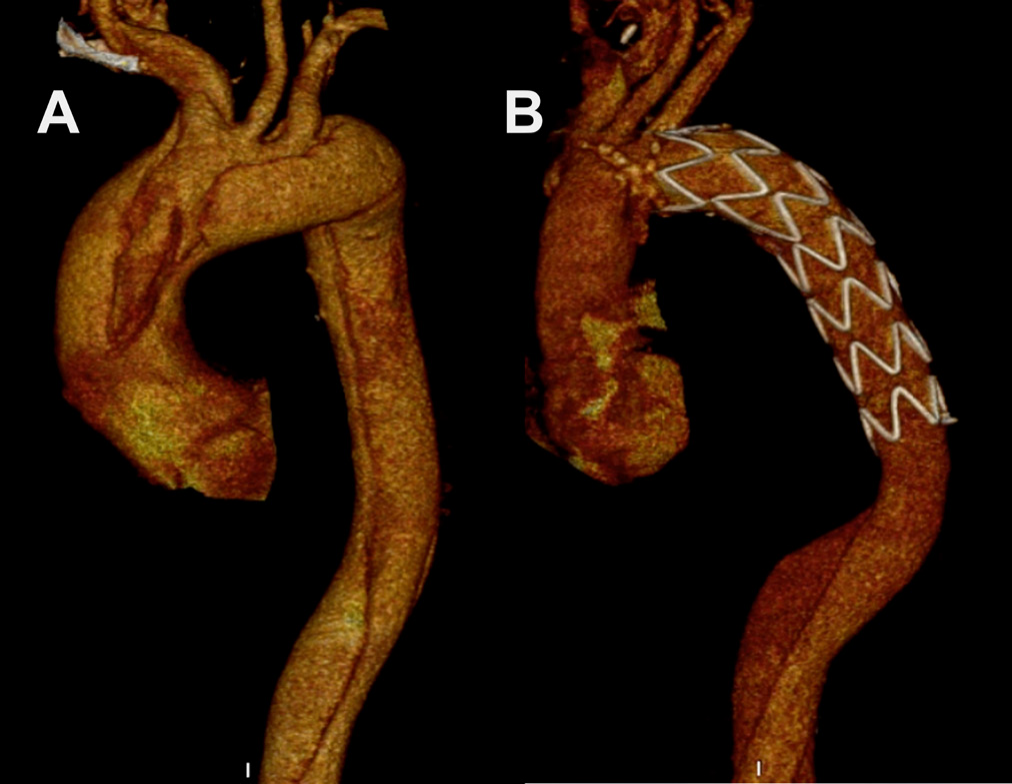
Preoperative (A) and postoperative (B) computerized tomography angiography images of frozen elephant trunk modification; zone 3, fixation with islet-shape arch repair.

Details of aortic and concomitant cardiac procedures are shown in Table 2. Patients with type B aortic dissections with ascending aorta and the aortic arch diameter with at least 40 mm or thrombosed false lumen extending into aortic arch treated with FET procedure were included in the study. Mean cardiopulmonary bypass (CPB), antegrade selective cerebral perfusion (ASCP) times, and visceral ischemia times were 194.5±66.5 minutes, 75.6±34.4 minutes, and 68.6±26.9 minutes, respectively. The Group A, which had the longer mean total perfusion time (225±82), also had the shorter mean duration of visceral ischemia (63.9±31.2 min) (*P*<0.001).

**Table 2:** Perioperative values.

	Overall (mean±SD)	Group A (mean±SD)	Group B (mean±SD)	*P*-value
Nasopharyngeal temperature (˚C)	25.8±16.3	25.9±1.4	25.7±1.7	0.32
Total perfusion time (min)	194.5±66.5	225±82	172.7±40.9	< 0.01*
ASCP time (min)	75.6±34.4	78.7±46.4	73.4±22.4	0.47
Visceral ischemia time (min)	68.6±26.9	63.9±31.2	71.9±23.1	0.03*
	**Overall, n (%)**	**Group A, n (%)**	**Group B, n (%)**	***P*-value**
LSA coverage	7(5)	3(5.2)	4(4.9)	0.62
Additional operation				
MR	4 (2.9)	0	4 (4.9)	0.14
Bentall	12 (8.6)	6 (10.3)	6 (7.4)	0.377
AVR	2 (1.4)	2 (3.4)	2 (2.5)	1
CABG	8 (5.8)	5 (8.6)	3 (3.7)	0.278
MVR	4 (2.9)	2 (3.4)	0	0.172

* Statistically significant

ASCP=antegrade selective cerebral perfusion; AVR=aortic valve replacement; CABG=coronary artery bypass grafting; LSA=left subclavian artery; MR=mitral reconstruction; MVR=mitral valve replacement; SD=standard deviation

### Statistical Analysis

In descriptive statistics, continuous mean ± standard deviation, median, minimum, and maximum values, and categorical data are given together with number and percentage values. In the statistical comparison of the data, Chi-square test, Fisher’s exact test, and likelihood ratio were appropriately used for categorical data. Fisher’s exact test and likelihood ratio test were used when the expected value in any of the cells in the probability tables evaluated was < 5. Testing distributions for normality for continuous data was assessed by Kolmogorov-Smirnov analysis, and one-way analysis of variance (ANOVA) and Kruskal-Wallis tests were used in the intergroup comparisons. *Post hoc* analyzes of meaningfulness between the zone groups were performed by one-way ANOVA Bonferroni *post hoc* test. After Kruskal-Wallis test, Mann-Whitney U test was used with Bonferroni correction. Survival was calculated by the Kaplan-Meier analysis and log rank (Mantel Cox) analysis was applied to the survival comparison in the zone groups. For statistical significance, a *P*-value < 0.05 at a 95% confidence interval was considered significant. Chi-square *post hoc* analyzes with Bonferroni correction was considered significant at values < 0.0167, and adjusted *P*-values were used in other *post hoc* assessments. For statistical analysis, IBM Corp. Released 2012, IBM SPSS Statistics for Windows, Version 21.0, Armonk, NY: IBM Corp. was used.

## RESULTS

### Early Results

Early mortality was observed in 20 (14.4%) patients, 12 of these (60%) had acute type A aortic dissection. These mortalities were due to bleeding (n=8), multiple organ failure (n=4), malperfusion (n=2), neurological complication (n=2), and low cardiac output (n=4). There was no statistical difference between groups in terms of early mortality rates (*P*=0,534).

Seven patients (5%) had permanent neurologic deficit (stroke and hemiplegia) and five patients (3.6%) had transient SCI (paraplegia and paraparesis). Two patients with paraplegia and three patients with paraparesis recovered completely with the aid of physiotherapy within a few months after surgery. Although the mean visceral ischemia time (81.2±14.2 min) was higher in patients with SCI, no significant difference was found between the patients with SCI and those without SCI (68.1±2.3 min) (*P*=0.41). Two patients died due to malperfusion syndrome with bowel and limb ischemia. The postoperative outcome data are given in Table 3.

**Table 3:** Postoperative outcomes.

	Overall, n (%)	Group A, n (%)	Group B, n (%)	*P*-value
In-hospital mortality	20 (14.4)	8 (13.8)	12 (14.8)	0,534
Total mortality	24 (17.3)	10 (17.2)	14 (17.3)	0.59
Re-intervention	14 (10.1)	4 (6.9)	10 (12.3)	0,22
Pulmonary complication	25 (18)	13 (22.4)	12 (14.8)	0,25
Dialysis (temporary/permanent)	18/2 (12.9/1.4)	7/1(12.1/1.7)	11/1 (13.6/1.2)	1/0.66
Stroke	7 (5)	4 (6.9)	3 (3.7)	0,32
Spinal cord ischemia	5 (3.6)	2 (3.4)	3 (3.7)	0.334
ICU stay (days), median (min-max)	5 (1-45)	5 (1-45)	3 (1-36)	0.3
Hospital stay (days), median (min-max)	13 (5-61)	12 (6-61)	11 (5-46)	0.56
Bleeding				
24 h	800 (100-5500)	700 (250-4000)	600 (100-5500)	0.75
Total	1400 (300-6000)	1100 (300-6000)	950 (300-5750)	0.7
Re-sternotomy (bleeding/tamponade)	16 (11.5)	7 (12.1)	9 (11.1)	0.86

ICU=intensive care unit

### Midterm Results

There were four late deaths at a mean follow-up time of 65.9±2.5 months. Two patients died from each of the groups. One patient of Group A treated for chronic type A aortic dissection was lost due to acute respiratory distress syndrome two years after surgery and the other Group A patient with chronic type B aortic dissection died two months after surgery due to post-dissection aneurysm rupture before scheduled thoracic endovascular repair (TEVAR). In Group B, one of the patients treated for acute type A aortic dissection died after reoperation for type 1A endoleak from the proximal suture line of FET stent graft due to stroke eight months after the first operation. The other Group B patient who had low cardiac ejection fraction due to congestive heart failure died 18 months after surgery.

During the follow-up period, TEVAR was performed in 12 patients as second stage therapy, due to extensive thoracic aortic aneurysm or post-dissection aneurysm (n=6) and stentinduced new entry (n=6). Two patients from Group B underwent supra-aortic de-branching with TEVAR due to type 1A endoleak from anastomotic suture line of FET stent graft. There was no difference between re-intervention and total mortality rates between the groups (Figures 1 and 2).

## DISCUSSION

Complex thoracic aortic diseases include pathologies bearing lethal complications, such as dissection or aneurysms, and have extensive aortic involvement, including ascending aorta, aortic arch, and descending aorta. Conventional surgery is challenging with high mortality and morbidity rates^[14]^. The description of new intervention models has been needed especially in the high-risk patient group for conventional surgery to reduce complications, prevent perioperative mortality, and cope with the challenges posed in major surgery in treatment of complex aortic diseases. Conventional elephant trunk technique defined by Borst^[15]^ pioneered the hybrid aortic surgery and with the improvement of the skeletonized grafts with stents, this technique has advanced into 'stented elephant trunk', 'open stent grafting', and FET procedures.

Over time, FET has undergone various modifications to facilitate the surgical technique based on the experience of the surgeon. However, decision-making is based on the extent of the disease and the concomitant pathology of the aortic arch. Western surgeons tend to perform hemiarch-like arch repair by preparing the arch vessels as an islet-shape and extending the graft to the small curvature in order to shorten the operation time. On the Asian side, total arch repair is completed with an additional fourth branch assist, which provides earlier distal body perfusion and warming during supra-aortic de-branching. Except for few authors^[3,11]^, fixation of the stent graft to the distal of the left subclavian artery is preferred as zone 3 (fixation zone 3), in order to provide longer segment stabilization of descending aorta.

Hospital mortality rates ranged from 0 to 17.2% in studies performed with fixation zone 3 without discrimination of arch replacement technique due to aortic involvement and comorbid factors in complex thoracic aortic diseases^[16]^. The pathology with the highest mortality rates was found to be acute type aortic dissections. When results were evaluated according to arch replacement, in-hospital mortality rates ranged from 0 to 12.8% in islet-shaped repair^[13,17-19]^, while in supra-aortic de-branching case series it was between 3,2% and 8.8%^[5,20,21]^. There are limited number of cases found in the English literature about the total arch de-branching with fixation zone 0. Hospital mortality rate of 3.2% was reported in the study of Shimimaru^[3]^ with 126 patients, and Havericht et al.^[11]^ found no mortality in their case series of 26 patients. It was observed that the zone selection and arch replacement technique did not make any difference in mortality between the groups in our study.

Unrelated to the surgical technique, FET procedure itself is a risk factor for neurological deficit development due to the presence of cervical vessels in the surgical field and its influence on spinal artery circulation. Using bilateral ASCP in moderate hypothermia to protect from cerebrovascular events, placement of the stent graft above the T8 level, keeping the ASCP time < 90 minutes in moderate hypothermia, and obtaining short durations of body circulatory arrest with early distal perfusion are well-accepted protection methods against SCI. Despite all protection methods, SCI and stroke rates for FET procedure range from 0.8% to 21.7% and 2.6% to 13%, respectively^[16]^. In our study, there were no differences in SCI and stroke rates between two groups, and the rates were consistent with the literature. These favorable results in our center are accomplished by relying on all protective measures during all FET procedures.

The aim of modified FET techniques is to improve the postoperative results by reducing the prevalence of postoperative renal failure and re-explorations for bleeding as well as providing technical facilitation^[9]^. The use of all techniques in a wide variety of patient groups has both perioperative and postoperative benefits, such as (i) shortened lower body circulatory arrest and hypothermia time, (ii) less spinal artery coverage, (iii) anastomotic simplicity and better bleeding control, and (iv) stabilization of dissection in cervical branches. Yet, it has its own disadvantages too, (i) the presence of un-resected aortic tissue may cause dilation in the future due to endoleaks, (ii) the occurrence of stroke risk due to supra-aortic de-branching, and (iii) risk of recurrent laryngeal nerve and phrenic nerve damage. However, in our study, there were no differences between the techniques in terms of postoperative outcomes as well as renal insufficiency, bleeding, and intensive care unit and hospital stays.

FET, described as a one-stage therapy especially for aortic dissections, reduces the rate of re-interventions providing shrinkage of aortic diameter by reducing the pressure on the aortic wall in aneurysms and including rapid false lumen thrombosis in dissections compared with conventional supracoronary graft replacement. However, post-dissection aneurysm and its complications required re-intervention due to reentries in the distal aorta. In our study, distal aortic re-interventions were performed because of stent-induced new entry (n=6) or scheduled secondary intervention thoracic aortic aneurysm or post-dissection aneurysm (n=6). Furthermore, in thoracic aortic aneurysms with mega aortic syndromes, it is possible to create a suitable landing zone for the second stage without any complications owing to the conventional elephant trunk graft^[22]^ and perform re-interventions safely without increasing the risk of surgery^[23]^. TEVAR was performed successfully in 12 patients without any complications in our study.

In our opinion, the fixation zone 0 and de-branching technique should be preferred in case of a second stage procedure requirement, such as presence of excessive number of distal reentries, in young and Marfan syndrome patients and in aneurysmatic aorta (initial aorta diameter > 40 mm) with a post-dissection aneurysm risk and extensive thoracic aortic aneurysms. However, it should not be forgotten that although shorter segment coverage of descending aorta is protective against spinal cord injury, this leads to poor spinal artery collateralization and may increase the risk during second stage intervention^[24]^. FET procedure by fixation zone 3 with islet-shape arch repair is suitable in cases when a concomitant valvular or coronary intervention is required, to decrease perfusion time and related complications.

In order to shorten the CPB duration, patients with left subclavian artery lateralization or sufficient posterior cerebral circulation may be treated with fixation zone 2 by coverage of the left subclavian artery as in TEVAR^[25]^. However, when islet-shaped arch repair is performed, there is a possibility of a second arch repair with a new supra-aortic de-branching due to enlargement of the remaining diseased aortic tissue. After all, FET offers a variety of surgical technique combinations specific to the patient and the pathology.

### Limitations

This is a retrospective cohort study in which complex thoracic aortic diseases were caused by various diseases and thus it was limited in terms of the inability to separate outcomes for each disease. There might have been a bias between groups due to complexity of aortic involvement which probably might affect surgical results. More accurate results will be obtained by enlargement of patient series and randomization of groups. Because of its advantageous aspects, such as surgical anastomotic facilities, early visceral perfusion, and stabilization of the cervical vessels, zone 0 fixation with supra-aortic de-branching is the preferred technique in our clinic.

## CONCLUSION

In conclusion, FET procedure, which makes the repair techniques of surgery more feasible in the treatment of complex aortic diseases, provides satisfactory early results. Personalized treatment should be applied according to the characteristics of each pathology and the patient's comorbid factors.

**Table:** 

Authors' roles & responsibilities	
MA	Substantial contributions to the conception or design of the work; or the acquisition, analysis, or interpretation of data for the work; drafting the work or revising it critically for important intellectual content; agreement to be accountable for all aspects of the work in ensuring that questions related to the accuracy or integrity of any part of the work are appropriately investigated and resolved; final approval of the version to be published
AA	Substantial contributions to the conception or design of the work; or the acquisition, analysis, or interpretation of data for the work; drafting the work or revising it critically for important intellectual content; final approval of the version to be published
OA	Substantial contributions to the conception or design of the work; or the acquisition, analysis, or interpretation of data for the work; final approval of the version to be published
AAD	Substantial contributions to the conception or design of the work; or the acquisition, analysis, or interpretation of data for the work; drafting the work or revising it critically for important intellectual content; final approval of the version to be published
ST	Substantial contributions to the conception or design of the work; or the acquisition, analysis, or interpretation of data for the work; final approval of the version to be published
DC	Substantial contributions to the conception or design of the work; or the acquisition, analysis, or interpretation of data for the work; drafting the work or revising it critically for important intellectual content; final approval of the version to be published
MS	Drafting the work or revising it critically for important intellectual content; final approval of the version to be published
MAT	Drafting the work or revising it critically for important intellectual content; final approval of the version to be published
